# Development of a Standardized Algorithm for Management of Newly Diagnosed Anorectal Malformations

**DOI:** 10.3390/children11040494

**Published:** 2024-04-20

**Authors:** Shruthi Srinivas, Alessandra Gasior, Sarah Driesbach, Natalie DeBacco, Liese C. C. Pruitt, Casey Trimble, Pooja Zahora, Claudia M. Mueller, Richard J. Wood

**Affiliations:** 1 Department of Colorectal and Pelvic Reconstructive Surgery, Nationwide Children’s Hospital, 611 E. Livingston Ave., Columbus, OH 43205, USA; 2 Department of Pediatric Surgery, Nationwide Children’s Hospital, Columbus, OH 43205, USA; 3 Department of Pediatric Surgery, Stanford Children’s Hospital, Stanford, CA 94304, USA

**Keywords:** neonatal intensive care unit, imperforate anus, cloacal malformation, diagnostic algorithm

## Abstract

Neonates with a new diagnosis of anorectal malformation (ARM) present a unique challenge to the clinical team. ARM is strongly associated with additional midline malformations, such as those observed in the VACTERL sequence, including vertebral, cardiac, and renal malformations. Timely assessment is necessary to identify anomalies requiring intervention and to prevent undue stress and delayed treatment. We utilized a multidisciplinary team to develop an algorithm guiding the midline workup of patients newly diagnosed with ARM. Patients were included if born in or transferred to our neonatal intensive care unit (NICU), or if seen in clinic within one month of life. Complete imaging was defined as an echocardiogram, renal ultrasound, and spinal magnetic resonance imaging or ultrasound within the first month of life. We compared three periods: prior to implementation (2010–2014), adoption period (2015), and delayed implementation (2022); *p* ≤ 0.05 was considered significant. Rates of complete imaging significantly improved from pre-implementation to delayed implementation (65.2% vs. 50.0% vs. 97.0%, *p* = 0.0003); the most growth was observed in spinal imaging (71.0% vs. 90.0% vs. 100.0%, *p* = 0.001). While there were no differences in the rates of identified anomalies, there were fewer missed diagnoses with the algorithm (10.0% vs. 47.6%, *p* = 0.05). We demonstrate that the implementation of a standardized algorithm can significantly increase appropriate screening for anomalies associated with a new diagnosis of ARM and can decrease delayed diagnosis. Further qualitative studies will help to refine and optimize the algorithm moving forward.

## 1. Introduction

Anorectal malformations (ARMs) present a unique challenge to the neonatal intensivist or pediatrician. ARMs occur across a spectrum from mild to severe, and while severe malformations may occasionally be identified during prenatal imaging, mild and moderate malformations are typically not, unless associated with significant congenital anomalies [1,2]. A patient newly diagnosed with an ARM at a low-volume community facility may require transfer to a large, experienced children’s hospital for further management [3]. Ultimately, the first 24–48 h of care for a newborn with ARM are critical, and may involve decisions such as how to screen for and manage associated congenital anomalies, whether a child needs a colostomy, and whether drainage of the bladder or vagina is necessary [4,5]. In addition, these first few days represent an important time in which to begin developing a therapeutic relationship with caregivers [6]. This relationship will likely extend beyond the immediate timeframe and may last for years as the child progresses through the stages of life with an ARM, including surgical interventions, bowel management programs, discussions of fertility and reproductive potential, and eventual transfer to adult care [7].

The neonatal period is a significant stress for any parent, but particularly those with a child with ARM [8]. The first few days of life for a family may generate more questions than answers, as this is the first time this diagnosis has been mentioned, but immediate high-level comprehensive evaluation is necessary. Diagnosis of ARM may be accompanied by diagnostic and prognostic uncertainty, in which the entirety of a diagnosis or prognosis is unknown or unable to be conveyed, which is known to be associated with high stress in parents with children in the neonatal intensive care unit (NICU) [9,10]. In children with ARM, the ARM is only part of the picture—associated diagnoses such as vertebral, cardiac, tracheoesophageal, renal, and limb are not uncommon [11]. Frequently grouped together and known as the VACTERL association, delayed diagnoses of these malformations can cause significant complications, including increased rates of renal failure in the case of missed hydronephrosis, soiling in the case of a missed tethered cord, and the need for intervention in the case of missed structural cardiac anomalies [8,12,13]. A delay in timely diagnosis may result in a need for more advanced workup and has potential to cause undue stress. Especially as the management of patients with ARM becomes preferentially referred to high-volume centers, providing structure to the workup and initial management of a patient newly diagnosed with ARM is necessary. Without this, there is a risk of delayed or incomplete diagnosis, undue stress for a family, and harm to the long-term therapeutic relationship with the medical community [14]. In our practice, as well as in the literature, we identified a need for a standardized algorithm to guide the workup of patients with newly diagnosed ARM [15]. While prior studies have highlighted short-term outcomes from the implementation of similar algorithms, there have been no institutional studies demonstrating the long-term success of these algorithms [16].

We aim to describe our institutional outcomes of a diagnostic algorithm for the initial workup and management of associated anomalies in a patient newly diagnosed with an ARM. We hypothesize that the algorithm will result in an increase in the complete imaging workup for patients with ARM. Ultimately, we hope that this algorithm can help to guide centers in managing the workup of newly diagnosed patients with ARMs to improve clinician–parent communication and decrease parental stress.

## 2. Materials and Methods

### 2.1. Study Overview

We performed a retrospective, single-site cohort study of children with newly diagnosed ARM undergoing any management at our institution within their first month of life. The algorithm was implemented in 2015 as part of the establishment of a dedicated pediatric colorectal center, including a multidisciplinary partnership with pediatric urology, pediatric and adolescent gynecology, and gastroenterology. The standardized approach to diagnosis and in-hospital management of ARM was defined at that time. In order to analyze the longitudinal success of the algorithm, three time periods were defined. Pre-implementation included patients managed prior to the algorithm implementation, defined as 2010–2014. The adoption phase of the algorithm was defined as patients managed under the algorithm during 2015. To determine the longitudinal success of the algorithm, a delayed post-implementation phase including the year 2022 was analyzed. The study was approved by our Institutional Review Board.

### 2.2. Algorithm Development

The development of the algorithm began with a focused literature review in which best practices regarding the initial workup of children with ARM were evaluated and combined with clinical experience and practical considerations to develop a short and easy-to-follow guide. The development of the algorithm was multidisciplinary and included contributions from pediatric colorectal surgeons, pediatric general surgeons, neonatal intensivists, radiologists, advance practice providers, social workers, and bedside nurses. The implementation of the algorithm involved standard education for neonatal and colorectal providers and directed feedback in rare settings of noncompliance. In addition to providing guidance on the appropriate imaging workup of associated anomalies and inpatient management, the algorithm also provided guidance for disclosing a diagnosis of ARM.

### 2.3. Participants

Patients diagnosed with an ARM presenting to our institution for management within one month of birth were included. Patients may have been born and undergone initial management at another institution, including appropriate imaging; however, if they were evaluated in their first month of life either in transfer to the NICU or as an outpatient, they remained included. Patients who presented after their first month of life were excluded, as their initial management was not comparable. Additionally, patients initially suspected to have an ARM that was ruled out upon further anatomic evaluation were also excluded. Cases of ARM were identified based on an institutional registry using appropriate International Classification of Diseases Ninth and Tenth codes.

### 2.4. Study Outcomes

For the purposes of analysis, the primary outcome was the fidelity with completed imaging, referred to as “complete midline workup”, and defined as the rate of completed imaging workup, including renal ultrasound, spinal ultrasound or magnetic resonance imaging (MRI), and echocardiogram. Imaging could be obtained at any institution but needed to be available to radiologists at our institution within the first month of life to be considered completed. Secondary outcomes included the incidence of any identified anomalies and delayed diagnosis of associated anomalies. An identified anomaly included any pathologic finding, regardless of severity, on any of the imaging modalities that required follow-up attention via surveillance for the development of symptoms, further imaging, referral to a specialist, or further intervention. These were summarized for all children regardless of the age of diagnosis in order to demonstrate a trend in the rates of associated diagnoses over time. Delayed diagnosis was defined as the identification of a spinal, renal, or cardiac anomaly using similar imaging to the screening modalities obtained after one month of age in a patient who was not screened within the first month of life. To compare the baseline characteristics of patients at each phase and ensure they were similar, a manual chart review for diagnosis was performed. Malformations were categorized as mild, moderate, severe, or unknown based on prior classifications (Supplemental Table S1) [17].

### 2.5. Statistical Analysis

Descriptive statistics, including counts and percentages, were calculated for all study variables. Data were compared across the three time periods, the pre-implementation phase, the adoption phase, and the delayed post-implementation phase, using chi square tests and Fisher’s exact tests as appropriate. For the secondary outcome of delayed diagnosis, only patients with a follow-up of at least five years were included in order to allow for an adequate time period in which associated diagnoses may be identified. All statistical analysis was performed using SAS version 8.1 (SAS Institute Inc., Cary, NC, USA).

## 3. Results

### 3.1. Algorithm

**Figure 1:**
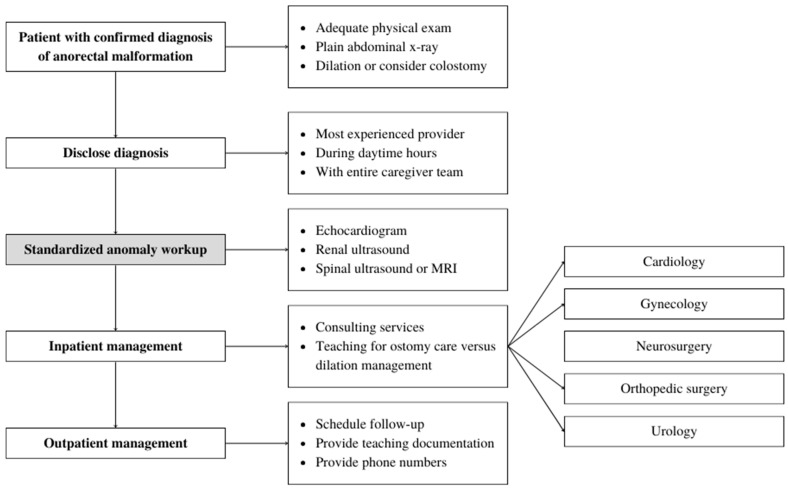
Standardized algorithm for the management of newly diagnosed anorectal malformations in our neonatal intensive care unit involves appropriate disclosure of the diagnosis, standardized workup of associated anomalies, inpatient management including stoma and dilation teaching, and outpatient follow-up and connections. To confirm the fidelity of the algorithm with respect to the workup of associated anomalies, the standardized anomaly workup, shaded in grey, was analyzed.

**Figure 2:**
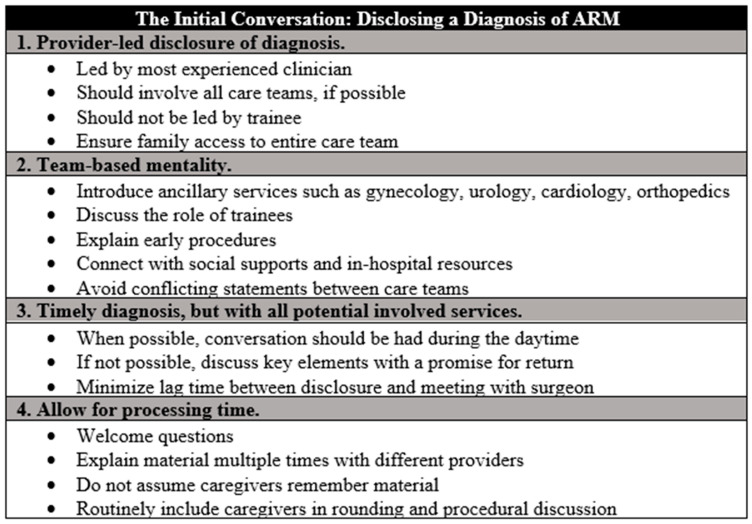
Our algorithm provides guidance for the initial disclosure of a diagnosis of anorectal malformation (ARM), including the provider leading the discussion, the team-based approach, timing of the disclosure, and processing time. This can be modified based on individual patient scenarios.

The resulting algorithm involves four phases: the initial diagnosis and disclosure of diagnosis, screening for associated comorbidities, inpatient management, and outpatient management (Figure 1). The algorithm begins with a confirmed diagnosis of ARM. The initial physical exam can be performed by any provider, but once suspicion of a possible ARM is generated, it should be confirmed by a provider experienced with ARM. This includes identifying the presence of a fistula. Good lighting is essential during this exam, and cotton-tip applicators and retraction of the labia may be necessary to correctly identify the anatomy. A plain abdominal X-ray is generally obtained as well. If a fistula is present, Hegar dilators should be inserted to size the fistula and clinical staff should perform serial Hegar dilations to allow for appropriate stool evacuation. If no fistula is present or the fistula is unable to be dilated, consideration should be given to a neonatal colostomy. For patients born in our NICU or those evaluated at a facility with easy patient transfer capabilities to our institution, the disclosure of a new diagnosis of ARM is particularly important, and guidance related to this was added to the medical algorithm guiding midline workup, as we feel that disclosure of the diagnosis and initiation of the therapeutic relationship is as important as the associated anomaly workup (Figure 2).

Guidance regarding the initial disclosure of the diagnosis of ARM was broken into four important components. After initial data are collected, we recommend that the person initiating conversation with the family regarding a new diagnosis is the most experienced provider available. This may be a surgeon, neonatologist, or pediatrician, but it should be whoever has the best ability to handle unexpected questions and assuage early concerns. Ideally, this person should not be a trainee, and rather should be a practicing physician who will remain part of the care team moving forward. Secondly, the algorithm provides guidance as to the role of the team. Care for the patient with a newly diagnosed ARM is multidisciplinary, and requires collaboration with many ancillary services, including gynecology, orthopedic surgery, urology, cardiology, and more. These teams may include a variety of practitioners from various levels. Introducing teams ahead of their arrival helps with unpredictability and ensures that the family is aware of why a consultant is seeing their child prior to their arrival. It also ensures that the care team limits conflicting statements between providers, which can erode the therapeutic relationship. Third, the algorithm recommends that the conversation surrounding a new diagnosis of ARM happens preferentially during daytime hours. This helps to ensure that everyone is rested, there is adequate time for conversation, and additional social support for the family can be present. While this may not be possible in the setting of an acute decompensation, the lag time between disclosure of diagnosis and meeting with the patient’s surgeon should be minimized. Finally, and potentially most importantly, families should be allowed processing time. This includes allowing for questions, even when out of scope of current management. All material, including diagnosis and consultant involvement, may need to be re-emphasized as families process and identify individuals who can help assist. Attention should be paid to the physical, emotional, and social effects.

Following the disclosure of diagnosis, the algorithm advances to the identification of associated anomalies, beginning with a complete midline workup. This includes a renal ultrasound, spinal MRI or ultrasound, and an echocardiogram prior to one month of age. Typically, these interventions are recommended while the patient is an inpatient after being born, but for those referred to us with a late diagnosis or transferred after diagnosis elsewhere, completion in the first month is required. In addition, physical exam for limb anomalies is performed, and evaluation for associated malformations such as hydrocolpos is performed on an as-needed basis. Finally, the algorithm provides guidance on inpatient management, including the need for operative interventions, the establishment of follow-up with appropriate services, and wound care teaching. For those patients that undergo diversion with a colostomy or ileostomy, teaching for management of the stoma is performed. For those with fistulae, dilation teaching is performed. Finally, prior to discharge, outpatient follow-up with colorectal surgery and appropriate consulting services is arranged. Teaching documentation is provided and phone numbers are given to allow for easy communication.

### 3.2. Evaluation of Algorithm

To evaluate the fidelity of our algorithm, we then proceeded to analyze measurable outcomes over the three predefined time periods. Specifically, we analyzed compliance with the standardized anomaly workup, as this represents both a vital and easily tracked component of the algorithm (Table 1). A total of 122 patients were evaluated, with 69 in the pre-implementation phase, 20 in the adoption phase, and 33 in the delayed post-implementation phase. There was no statistically significant difference in the rates of diagnosis of each subtype of malformation by phase of implementation, including mild (63.8% vs. 60.0% vs. 60.6%), moderate (4.4% vs. 5.0% vs. 12.1%), severe (14.5% vs. 25.0% vs. 18.2%), and unknown (17.4% vs. 10.0% vs. 9.1%, overall *p* = 0.63). Prior to the implementation of the algorithm, 65.2% of patients had a complete midline workup within the first month of life. During implementation, this reduced to 50%; however, it increased significantly to 97.0% in the delayed post-implementation phase (*p* = 0.0003). Rates of success were most significant for spinal MRI and ultrasound (71.0% vs. 90.0% vs. 100.0%, *p* = 0.01), though they were also significant for echocardiogram (82.6% vs. 50.0% vs. 97.0%, *p* = 0.0001). The incidence of completed renal ultrasounds was not significantly different before and after implementation (95.7% vs. 100.0% vs. 100.0%, *p* = 0.31), nor were the rates of identified anomalies in any organ system, indicating stable association between ARM and other comorbidities over time (95.7% vs. 95.0% vs. 90.9%, *p* = 0.36). In comparing those with follow-up of at least five years, there was a significantly higher incidence of delayed diagnosis in the pre-implementation group (47.6% vs. 10.0%, *p* = 0.05). Overall fidelity with the algorithm at the delayed post-implementation phase was 97.0%.

## 4. Discussion

In this single-institution, long-term follow-up study, we demonstrate that a short, easy-to-follow algorithm developed by a multi-disciplinary team for patients with ARM can significantly increase the completion of the recommended screening process for a midline workup and is associated with a decrease in delayed diagnosis. This suggests the algorithm could be applied to low-volume centers to ensure that patients with ARM undergo appropriate screening and management at all institutions.

Identifying associated anomalies in children with ARM is critically important, as it informs the short- and long-term prognosis and provides guidance regarding the need for intervention prior to surgical management of the ARM [4]. For example, in patients diagnosed with VACTERL association in addition to their ARM, the coexistence of VACTERL anomalies negatively affects both surgical outcomes and bowel function later in life [18]. Patients with a delayed diagnosis of a tethered cord may experience higher rates of fecal and urinary incontinence [19]. Because the risk of delay is so significant, the current recommendation is therefore for a comprehensive midline workup in patients with ARM [20]. While the specifics of complete screening vary by institution and by country, most recommendations include a vertebral X-ray or spinal cord ultrasound, an echocardiogram, a renal ultrasound, and potentially a genetics workup based on clinical presentation [21]. Rates of anomalies are reported at as high as 31% for urologic anomalies, 10% for cardiac anomalies, and 8% for spinal cord anomalies, even in the absence of a unifying diagnosis such as VACTERL association [22]. Despite the high incidence of these anomalies and potential consequences of missed diagnosis, large, multi-institutional studies show compliance with complete screening as low as 11.3% [23]. Effective algorithmic management is therefore necessary.

Our institutional algorithm defines completed screening as including an echocardiogram, renal ultrasound, and spinal MRI or ultrasound. This was chosen due to the relatively high incidence of associated anomalies within these systems as well as the potential for an identified anomaly in one of these systems to result in a significant change to the clinical plan. Typically, we pursue these screenings in the first few days of life in the NICU. Depending on the severity of the anomaly, consultations with cardiology, urology, nephrology, and neurosurgery may be required. In addition to early knowledge and management, early screening also means early disclosure to the family. Given the stress at the time of diagnosis of an ARM and the potential for uncertainty related to long-term outcomes, it is important to provide families with as complete of a diagnostic picture as possible.

Unsurprisingly, our data demonstrate a high compliance with renal ultrasounds before, during, and after the implementation of the standard diagnosis algorithm. The association between genitourinary anomalies and ARM is well established, and may have led to increased screening prior to formal implementation of the algorithm [24]. Additionally, renal anomalies may be more frequently diagnosed prenatally, increasing compliance with postnatal evaluation [25]. Interestingly, rates of compliance with echocardiograms fell during the adoption phase of the algorithm. While the precise reason for this is unclear, this change was during the initial phase of implementation. During this time, our colorectal center was being established, which resulted in an increase in complex referrals. We may have failed to adequately obtain performed echocardiograms from outside institutions during this time, or patients may have been managed at other facilities without screening algorithms with subsequent referral. Despite this, it is encouraging that at the delayed post-implementation phase, a significant improvement in compliance with screening echocardiogram could be observed, as screening of the cardiovascular system is of particular importance to both systems prior to considering surgical interventions [26]. Finally, adherence to spinal MRI and ultrasound significantly improved to 100% of children in the delayed post-implementation phase. Given the association between ARM, tethered cord, and future continence potential, this is extremely encouraging. Interestingly, during the immediate post-implementation phase, the rates of spinal MRI were highest as compared to spinal ultrasound. This may reflect the referral pattern, with outside institutions obtaining MRIs during this time and transferring patients to our care or may reflect improvement in screening spinal ultrasound over time at our institution. More than simply ensuring that screening is completed, we hoped to appropriately identify anomalies in our population, preventing a delay in diagnosis. In our patients, the rate of missed diagnosis decreased significantly between pre-implementation and post-implementation. Importantly, and in contrast to other work, the algorithm has shown high fidelity over almost eight years of implementation without significant change, representing significant improvement over the estimate of 11.3%, which potentially excludes centers without a specialized colorectal facility [23]. This suggests that it is both easy to follow and effective in accomplishing its intended purpose, which highlights its generalizability to other institutions looking to make similar changes.

It is essential to recognize that while standardized algorithms are important tools for the clinician, they risk the potential for a loss of empathy and compassion in the face of the use of a one-size-fits-all methodology [27]. While there are multiple studies that highlight the medical benefit to utilizing screening algorithms, few works highlight the impact that using these tools may have on a family [28,29]. In fact, many algorithms end with the caveat that they should be amended to address individual situations whenever necessary. Therefore, our algorithm was structured to provide guidance regarding the initial disclosure of the diagnosis of ARM, as this is a profoundly important time period in which the therapeutic relationship can be built with the family, ensuring trust in the medical system moving forward. Specifically, we recommend the disclosure of diagnosis of a new ARM comes from the most experienced provider on the team, whether that be the neonatal intensivist or the colorectal surgeon. While trainees such as residents and fellows are undoubtedly important members of the team, by ensuring the availability of a senior member of the team, we feel families receive accurate information from someone who may be expected to become a familiar face moving forward. In addition to discussing the diagnosis, we find it helpful for the anticipated initial hospital course to be discussed with families. The midline workup should be discussed, including the need for the involvement of consulting services. By providing transparency to the need for midline workup and the potential for identification of anomalies, we feel that families may be more comfortable asking questions, understanding why certain investigations are being pursued, and developing individual empowerment. Finally, prior studies within other methodologies demonstrate that in families learning about a critical diagnosis, multiple forms of educational interventions need to be developed, with frequent re-evaluation to confirm that families are adequately processing information as it is being delivered [30]. For this reason, we work closely with our families to ensure that they have adequate time at clinic appointments in the early phase, access to surgeons for questions as they develop, and resources upon discharge about where to find more information on their diagnosis, support groups, and access to the clinical team post-discharge.

This is one of the first studies to implement a standardized protocol for screening for associated anomalies in patients with ARM, but it is subject to several limitations. As it was a retrospective review over a prolonged period in which several changes were made, it is unclear what additional processes or discussions may have affected compliance with recommended screening during the implementation of the algorithm. It is surprising that the completion of echocardiograms declined during the adoption phase; however, it is more reassuring that this significantly improved during the post-implementation period. Additionally, there is a higher rate of overall reported anomalies in our cohort compared to other descriptions in the literature, with quoted rates being as high as 78% [31]. This may be because our institution is a major, quaternary referral center that receives a higher percentage of complicated patients requiring expert management; this is evident in the breakdown of diagnosis per group, showing that almost one-fifth of our population presented with a severe ARM. While we can assess the rates of delayed diagnosis in our cohort, it is unclear what the impact of a delay in diagnosis was on a patient and their family in terms of workup, management, and the need for further intervention. In generating a definition for “complete workup”, we chose to include an echocardiogram, a spinal MRI or ultrasound, and a renal ultrasound. As knowledge surrounding ARM advances, recommendations for screening may continually change. For example, anomalies such as hydrocolpos, presacral masses, and reflex nephropathy may not necessarily have been adequately identified with the current algorithm and may be considered moving forward. Finally, the algorithm we developed strives to not only address imaging but also to appropriately manage the initial disclosure of a diagnosis of ARM, the inpatient teaching and education, and connections to the outpatient team. This is more difficult to quantitatively study and represents an area of future research in demonstrating the fidelity of the entire algorithm.

## 5. Conclusions

In a single center, we demonstrate that the implementation of a standardized protocol guiding cardiac, renal, and spinal screening in patients with ARM can be effective over a prolonged period, with high fidelity and high completion rates. Further qualitative work on the impact of the algorithm on patients and providers will help to refine and optimize it for application in lower-volume centers.

## Figures and Tables

**Table 1:** Results of application of diagnosis algorithm in the pre-implementation, adoption, and post-implementation phases. Emboldened *p*-values are significant.

	Pre-Implementation (2010–2014) *n* = 69	Adoption Phase (2015) *n* = 20	Post-Implementation (2022) *n* = 33	*p*-Value
Complete imaging workup ^a^	45 (65.2)	10 (50.0)	32 (97.0)	**0.0003**
Echocardiogram	57 (82.6)	10 (50.0)	32 (97.0)	**0.0001**
Renal ultrasound	66 (95.7)	20 (100.0)	33 (100.0)	0.31
Spinal MRI/US	49 (71.0)	18 (90.0)	33 (100.0)	**0.001**
*MRI*	45 (91.8)	12 (66.7)	32 (97.0)	**0.006**
*US*	4 (8.1)	6 (33.3)	1 (3.0)	
Any identified abnormality	66 (95.7)	19 (95.0)	30 (90.9)	0.36
Delayed diagnosis of associated anomaly ^b^	10 (47.6)	1 (10.0)	-	**0.05**

^a^: defined as completed renal ultrasound, spinal MRI or ultrasound, and echocardiogram within one month of age. ^b^: includes only patients with follow-up.

## Data Availability

The data presented in this study are available upon request from the corresponding author due to privacy reasons.

## Supplementary Materials

The following supporting information can be downloaded at: https://www.mdpi.com/article/10.3390/children11040494/s1, Table S1: Anorectal Malformation Severity.
