# Supplementary material for: Development of a Standardized Algorithm for Management of Newly Diagnosed Anorectal Malformations
Source: Children (Basel). 2024 Apr 20;11(4):494. doi: 10.3390/children11040494 (PMC11049553; doi:10.3390/children11040494)
Supplement: Supplementary file 1 [file children-11-00494-s001.zip › children-2947841-supplementary.pdf]

Supplemental Table S1. Anorectal Malformation Severity.

Mild	Rectoperineal fistula
	Rectovestibular fistula
	H-type fistula
	Anal stenosis
	Rectal stenosis
	Rectal atresia
Moderate	Cloaca (<3 cm)
	Rectovaginal fistula
Severe	Cloaca (>3 cm)
	Posterior cloacal variant
	Rectovesical fistula
	Cloacal exstrophy
Unknown	Other
	Unknown
	No fistula
	Rectourethral fistula, unknown level
